# Supplementary material for: ­Complete plastid genome sequences of two species of the Neotropical genus Brunellia (Brunelliaceae)
Source: PeerJ. 2020 Jan 28;8:e8392. doi: 10.7717/peerj.8392 (PMC6993752; doi:10.7717/peerj.8392)
Supplement: Supplemental Information 1 [file peerj-08-8392-s001.pdf]

SUPLEMENTARY MATERIAL

Complete plastid genome sequences of two species of the Neotropical genus *Brunellia* (Brunelliaceae)

Table S1. Sampling used for the phylogenetic analyses.

Table S2. Chloroplast genome features of *Brunellia antioquensis* and *B. trianae*

Table S3. Plastid genes found in *Brunellia antioquensis* and *B. trianae*

Table S4. Lengths of exons and introns for the split genes on *Brunellia antioquensis* and *B. trianae* plastomes.

Table S5. List of repeated sequences in the plastome of *Brunellia antioquensis*.

Table S6. List of repeated sequences in the plastome of *Brunellia trianae*.

Table S7. Nucleotide substitutions and InDels in the plastomes of *Brunellia antioquensis* and *B. trianae*

Table S8. Phylogenetic studies that support each one of the three possible topologies among the COM clade.

Fig. S1. Plastid genome alignment from Mauve among selected COM representatives.

Fig. S2. Plastid genome alignment from mVISTA among all available sequences of COM species.

Table S1. Sampling used for the phylogenetic analyses.

Taxa included in the phylogenetic analysis. The samples used in the plastid genome comparison are indicated.

Clade	Order APG 2009	Family	Species	Source	GenBank Accession	Plastid genome comparison	Original publication
FABIDS	Zygophyllales	Zygophyllaceae	*Bulnesia arborea* Jacq. Engl.	Ruhfel et al., 2014a	EU002159, EU002172, EU002205, EU002275, EU002299, EU002388, EU002478, GQ998005-GQ998073		Wang et al., 2009; Moore et al., 2010
FABIDS	Zygophyllales	Zygophyllaceae	*Larrea tridentata* DC. Coville	GenBank	KT272174		Unpublished
FABIDS	Zygophyllales	Zygophyllaceae	*Tetraena mongolica* Maxim.	GenBank	MH325021		Unpublished
FABIDS/COM	Celastrales	Celastraceae	*Euonymus japonicus* Thunb.	GenBank	KP189362	X	Choi & Park, 2016
FABIDS/COM	Celastrales	Celastraceae	*Parnassia brevistyla* Brieger Hand.-Mazz.	GenBank	MG792145	X	Xia et al., 2018
FABIDS/COM	Celastrales	Rhamnaceae	*Catha edulis* Vahl Forssk. ex Endl.	GenBank	KT861471	X	Gu et al., 2018
FABIDS/COM	Malpighiales	Euphorbiaceae	*Jatropha curcas* L.	Ruhfel et al., 2014a	FJ695500		Asif et al., 2010
FABIDS/COM	Malpighiales	Euphorbiaceae	*Manihot esculenta* Crantz	GenBank	EU117376	X	Daniell et al., 2008
FABIDS/COM	Malpighiales	Euphorbiaceae	*Ricinus communis* L.	Ruhfel et al., 2014a	JF937588		Rivarola et al., 2011
FABIDS/COM	Malpighiales	Passifloraceae	*Passiflora biflora* Lam.	Ruhfel et al., 2014a	EU017064-EU017138		Jansen et al., 2007

FABIDS/COM	Malpighiales	Salicaceae	*Populus alba* L.	Ruhfel et al., 2014a	AP008956		Okumura et al., 2006
FABIDS/COM	Malpighiales	Salicaceae	*Salix babylonica* L.	GenBank	KT449800	X	Wang & Yang, 2016
**FABIDS/COM**	**Oxalidales**	**Brunelliaceae**	***Brunellia antioquensis* Cuatrec. Cuatrec.**	**This study**		**X**	**This study**
**FABIDS/COM**	**Oxalidales**	**Brunelliaceae**	***Brunellia trianae* Cuatrec.**	**This study**		**X**	**This study**
FABIDS/COM	Oxalidales	Cunoniaceae	*Ceratopetalum apetalum* D.Don	GenBank	KT716495	X	Foster et al., 2016
FABIDS/COM	Oxalidales	Elaeocarpaceae	*Sloanea australis* F.Muell.	GenBank	KT716504	X	Foster et al., 2016
FABIDS/COM	Oxalidales	Oxalidaceae	*Averrhoa carambola* L.	GenBank	KU569488	X	Jo et al., 2016
FABIDS/COM	Oxalidales	Oxalidaceae	*Oxalis latifolia* Kunth	Ruhfel et al., 2014a	EU002165, EU002186, EU002248, EU002282, EU002350, EU002438, EU002528, GQ998511-GQ998580		Wang et al., 2009; Moore et al., 2010
FABIDS/ N-fixing clade	Cucurbitales	Cucurbitaceae	*Cucumis melo* L.	GenBank	JF412791		Rodríguez-Moreno et al., 2011
FABIDS/ N-fixing clade	Fabales	Fabaceae	*Arachis hypogaea* L.	GenBank	KJ468094		Schwarz et al., 2015
FABIDS/ N-fixing clade	Fabales	Fabaceae	*Glycine max* L.Merr.	Ruhfel et al., 2014a	DQ317523		Saski et al., 2005
FABIDS /N-fixing clade	Fagales	Fagaceae	*Castanea mollissima* Blume	GenBank	KY951992		Cheng et al., 2018

FABIDS/ N-fixing clade	Fagales	Fagaceae	*Quercus nigra* L.	Ruhfel et al., 2014a	EU002167, EU002188, EU002254, EU002284, EU002357, EU002445, EU002535, GQ998653-GQ998724	Wang et al., 2009; Moore et al., 2010
FABIDS/ N-fixing clade	Rosales	Moraceae	*Ficus racemosa* L.	GenBank	KT368151	Mao & Bi, 2016
FABIDS/ N-fixing clade	Rosales	Moraceae	*Morus indica* L.	Ruhfel et al., 2014a	DQ226511	Ravi et al., 2006
FABIDS/ N-fixing clade	Rosales	Rosaceae	*Pentactina rupicola* Nakai	Ruhfel et al., 2014a	JQ041763	Kim & Kim, 2016
FABIDS/ N-fixing clade	Rosales	Rosaceae	*Prunus persica* L. Batsch	GenBank	HQ336405	Jansen et al., 2011
MALVIDS	Brassicales	Brassicaceae	*Arabidopsis thaliana* L. Heynh.	Ruhfel et al., 2014a	AP000423	Sato, 1999
MALVIDS	Brassicales	Brassicaceae	*Arabis alpina* L.	GenBank	HF934132	Melodelima & Lobréaux, 2013
MALVIDS	Brassicales	Caricaceae	*Carica papaya* L.	Ruhfel et al., 2014a	EU431223	Unpublished
MALVIDS	Crossosomatales	Staphyleaceae	*Staphylea colchica* Steven	Ruhfel et al., 2014a	EU002168, EU002189, EU002261, EU002285, EU002364, EU002453, EU002543, GQ998725-GQ998796	Wang et al., 2009; Moore et al., 2010
MALVIDS	Geraniales	Geraniaceae	*Pelargonium quercifolium* L. f. L'Hér.	GenBank	KM527897	Weng, Ruhlman & Jansen, 2017

MALVIDS	Geraniales	Vivianiaceae	*Viviania marifolia* Cav.	GenBank	KF240615	Weng et al., 2014
MALVIDS	Malvales	Malvaceae	*Aquilaria yunnanensis* S.C.Huang	GenBank	MG656407	Zhang et al., 2019
MALVIDS	Malvales	Malvaceae	*Gossypium arboreum* L.	Ruhfel et al., 2014a	HQ325740	Xu et al., 2012
MALVIDS	Malvales	Malvaceae	*Heritiera angustata* Pierre	GenBank	MG897126	Zhao et al., 2018
MALVIDS	Myrtales	Myrtaceae	*Eucalyptus grandis* W.Hill	GenBank	HM347959	Paiva et al., 2011
MALVIDS	Myrtales	Onagraceae	*Oenothera parviflora* L.	Ruhfel et al., 2014a	EU262891	Greiner et al., 2008
MALVIDS	Sapindales	Anacardiaceae	*Spondias tuberosa* Arruda ex Koster	GenBank	KU756562	Santos et al., 2019
MALVIDS	Sapindales	Rutaceae	*Citrus sinensis* L. Osbeck	Ruhfel et al., 2014a	DQ864733	Bausher et al., 2006
SUPERROSIDS	Saxifragaceae	Altingiaceae	*Liquidambar styraciflua* L.	Ruhfel et al., 2014a	EU002164, EU002182, EU002239, EU002281, EU002341, EU002429, EU002519, GQ998438-GQ998510	Wang et al., 2009; Moore et al., 2010
SUPERROSIDS	Saxifragaceae	Saxifragaceae	*Heuchera sanguinea* Engelm.	Ruhfel et al., 2014a	EU002163, EU002180, EU002228, EU002280, EU002327, EU002415, EU002506, GQ998365-GQ998437	Wang et al., 2009; Moore et al., 2010
SUPERROSIDS	Vitales	Vitaceae	*Vitis acerifolia* Raf.	GenBank	LC317004	Zecca et al., 2019

Table S2. Chloroplast genome features of *Brunellia antioquensis* and *B. trianae*

	B. antioquensis		*B. trianae*	
	bp (%)	GC (%)	bp (%)	GC (%)
rRNA	9,050 (5.73%)	55.45	9,050 (5.73%)	55.45
tRNA	2,927 (1.85%)	52.75	2,927 (1.85)	52.75
proteins coding regions	82,209 (52.13%)	37.89	82,218 (52.14%)	37.89
Intergenic regions	64,140 (40.67%)	32.84	64,208 (40.71%)	32.84
Introns	16,289 (10.33%)	34.9	16,289 (10.32%)	34.9

Table S3. Plastid genes found in *Brunellia antioquensis* and *B. trianae*

Category	Groups of gene	Name of genes
Protein synthesis and DNA-replication	Transfer RNAs	*trnA-UGC*(× 2), *trnC-GCA*, *trnD-GUC*, *trnE-UUC*, *trnF-GAA, trnfM-CAU, trnG-GCC, trnG-UCC, trnH-GUG, trnI-CAU*(× 2), *trnI-GAU*(× 2), *trnK-UUU, trnL-CAA*(× 2), *trnL-UAA, trnL-UAG, trnM-CAU, trnN-GUU*(× 2), *trnP-UGG, trnQ-UUG, trnR-ACG*(× 2), *trnR-UCU, trnS-GCU, trnS-GGA, trnS-UGA, trnT-GGU, trnT-UGU, trnV-GAC*(× 2), *trnV-UAC*(× 2), *trnW-CCA, trnY-GUA*
	Ribosomal RNAs	*rrn16*(× 2)*, rrn23*(× 2), *rrn4.5*(× 2), *rrn5*(× 2)
	Ribosomal protein small subunit	*rps2, rps3, rps4, rps7*(× 2), *rps8, rps11, rps12*(× 3), *rps14, rps15, rps16, rps18, rps19*
	Ribosomal protein large subunit	*rpl2*(× 2)*, rpl14, rpl16, rpl20, rpl22, rpl23*(× 2), *rpl33, rpl36*
	Subunits of RNA polymerase	*rpoA, rpoB, rpoC1, rpoC2*
Photosynthesis	Photosystem I	*psaA, psaB, psaC, psaI, psaJ*
	Photosystem I assembly protein	*ycf4*
	Photosystem II	*psbA, psbB, psbC, psbD, psbE, psbF, psbH, psbI, psbJ, psbK, psbL, psbM, psbN, psbT, psbZ*
	Cythochrome b/f complex	*petA*, *petB, petD*, *petG, petL petN*
	ATP synthase	*atpA, atpB, atpE, atpF, atpH, atpI*
	NADH-dehydrogenase	*ndhA, ndhB*(× 2)*, ndhC, ndhD, ndhE, ndhF, ndhG, ndhH, ndhI*, *ndhJ*, *ndhK*
	Large subunit Rubisco	*rbcL*
Miscellaneous group	Acetyl-CoA carboxylase	*accD*
	Cytochrome c biogenesis	*ccsA*
	Inner membrane protein	*cemA*
	ATP-dependent protease	*clpP*
	Translation initiation factor	*infA*
	Maturase	*matK*
	Translocon at the inner membrane	*ycf1*
	Enhancing stress tolerance	*ycf3*
Gene unknown function		*ycf2*(× 2)*, ycf15*(× 2)
Pseudogene unknown function	Conserved hypothetical chloroplast ORF	*ψycf1*

Table S4. Lengths of exons and introns for the split genes on *Brunellia antioquensis* and *B. trianae* plastomes.

Variations of *B. trianae* are in parentheses.

Gene Name	Region	Exon I (bp)	Intron I (bp)	Exon II (bp)	Intron II (bp)	Exon III (bp)	Total size
rps16	LSC	42	919	230			1,191
atpF	LSC	411	722	144			1,277
rpoC1	LSC	1619	754	430			2,803
ycf3	LSC	153	691(692)	228	736	126	1,935(1,934)
clpP	LSC	228	618(617)	292	834	71	2,042(2,043)
petB	LSC	9	779	648			1,436
petD	LSC	8	710	475			1,193
rpl16	LSC	397	1036	12			1,445
rpsl2	IR	434	659	397			1,490
rps12	LSC	114	-	27	539	231	
ndhB	IR	756	679	777			2,212
ndhA	SSC	558	1,102	540			2,200
trnK-UUU	LSC	35	2,549	37			2,621
trnG-UCC	LSC	23	744	48			815
trnL-UAA	LSC	37	544	50			631
trnV-UAC	LSC	37	593	39			669
trnI-GAU	IR	42	938	35			1,015
trnA-UGC	IR	38	802	35			875

Table S5. List of repeated sequences in the plastome of *Brunellia antioquensis*.

C: complement match, F: forward (direct) match, P: palindromic match, R: reverse match.

	Repeat A			Repeat B			
Repeat length (bp)	Star site	Location	Region	Star site	Location	Region	Repeat type
30	8,544	*trnS-GCU*	LSC	47,779	*trnS-GCU*	LSC	P
30	41,110	*psaB*	LSC	43,334	*psaA*	LSC	F
28	33,878	intergenic (*trnT-GGU_psbD*)	LSC	33,891	intergenic (*trnT-GGU-psbD*)	LSC	F
24	128,836	intergenic (*trnL-UAG-ndhF*)	SSC	128,860	intergenic (*trnL-UAG-ndhF*)	SSC	F
23	66,708	intergenic (*petA-psbJ*)	LSC	66,748	intergenic (*petA-psbJ*)	LSC	P
23	94,637	*ycf2*	IRB	94,673	*ycf2*	IRB	F
23	94,637	*ycf2*	IRB	150,384	*ycf2*	IRA	P
23	94,673	*ycf2*	IRB	150,420	*ycf2*	IRA	P
23	150,384	*ycf2*	IRA	150,420	*ycf2*	IRA	P
22	4,901	intergenic (*matK-rps16*)	LSC	9,205	intergenic (*trnS-GCU-trnR-UCU*)	LSC	P
22	10,448	intergenic (*trnG-UCC-trnR-UCU*)	LSC	10,475	intergenic (*trnG-UCC-trnR-UCU*)	LSC	P
22	17,397	intergenic (*rps2-rpoC2*)	LSC	72,053	intergenic (*rpl20-clpP*)	LSC	R
21	8,550	*trnS-GCU*	LSC	37,852	*trnS-UGA*	LSC	F
21	9,203	intergenic (*trnS-GCU-trnG-UCC*)	LSC	125,118	intergenic (*psaC-ndhD*)	SSC	R
21	9,203	intergenic (*trnS-GCU-trnG-UCC*)	LSC	125,118	intergenic (*psaC-ndhD*)	SSC	F
21	10,622	intergenic (*trnS-GCU-trnG-UCC*)	LSC	85,637	*rpl16*	LSC	C
21	37,852	*trnS-UGA*	LSC	47,782	*trnS-GGA*	LSC	P
21	53,251	*ndhK*	LSC	59,847	Intergenic (*rbcL-accD*)	LSC	P
21	94,609	*ycf2*	IRB	150,360	*ycf2*	IRA	P
21	94,699	*ycf2*	IRB	150,450	*ycf2*	IRA	F
21	102,391	intergenic (*rps7-trnV-GAC*)	IRB	142,668	intergenic (*trnV-GAC-rps7*)	IRA	C
21	150,360	*ycf2*	IRB	150,450	*ycf2*	IRA	F
20	5,543	intergenic (*matK-rps16*)	LSC	13,886	intergenic (*atpF-atpH*)	LSC	R
20	10,624	intergenic (*trnS-GCU-trnR-UCU*)	LSC	57,156	intergenic (*atpB-rbcL*)	LSC	P
20	13,216	*atpF*	LSC	74,489	*clpP*	LSC	R
20	37,920	*trns-UGA*	LSC	47,721	*trnS-GGA*	LSC	P

Table S6. List of repeated sequences in the plastome of *Brunellia trianae*.

C: complement match, F: forward (direct) match, P: palindromic match, R: reverse match.

	Repeat A			Repeat B			
Repeat length (bp)	Star site	Location	Region	Star site	Location	Region	Repeat type
88	30,289	intergenic (*petN-psbM*)	LSC	30,311	intergenic (*petN-psbM*)	LSC	F
66	30,289	intergenic (*petN-psbM*)	LSC	30,333	intergenic (*petN-psbM*)	LSC	F
44	30,289	intergenic (*petN-psbM*)	LSC	30,355	intergenic (*petN-psbM*)	LSC	F
30	8,525	*trnS-GCU*	LSC	47,831	*trnS-GGA*	LSC	P
30	41,161	*psaB*	LSC	43,385	*psaA*	LSC	F
28	33,943	intergenic (*trnT-GGU-psbD*)	LSC	33,956	intergenic (*trnT-GGU-psbD*)	LSC	F
24	17,380	intergenic (*rps2-rpoC2*)	LSC	72,122	intergenic (*rpl20-clpP*)	LSC	C
24	128,913	intergenic (*trnL-UAG-ndhF*)	SSC	128,937	intergenic (*trnL-UAG-ndhF*)	SSC	F
23	9,182	intergenic (*trnS-GCU-trnR-UUC*)	LSC	17,377	intergenic (*rps2-rpoC2*)	LSC	F
23	66,778	intergenic (*petA-psbj*)	LSC	66,818	intergenic (*petA-psbj*)	LSC	P
23	94,710	*ycf2*	IRB	94,746	*ycf2*	IRB	F
23	94,710	*ycf2*	IRB	150,461	*ycf2*	IRA	P
23	94,746	*ycf2*	IRB	150,497	*ycf2*	IRA	P
23	150,461	*ycf2*	IRA	150,497	*ycf2*	IRA	F
22	10,429	intergenic (*trnS-GCU-trnR-UCU*)	LSC	10,456	intergenic (*trnS-GCU-trnR-UCU*)	LSC	P
22	30,289	intergenic (*petN-psbM*)	LSC	30,377	intergenic (*petN-psbM*)	LSC	F
21	8,531	*trnS-GCU*	LSC	37,917	*trnS-UGA*	LSC	F
21	37,917	*trnS-UGA*	LSC	47,834	*trnS-GGA*	LSC	P

Table S7. Nucleotide substitutions and InDels in the plastomes of *Brunellia antioquensis* and *B. trianae*

Polymorphism	Length (bp)	Motif	Marker	Location	Region
InDel	1	A	*matK-trnK*	intergenic	LSC
InDel	1	A	*trnK-rps16*	intergenic	LSC
InDel	1	A	*trnK-rps16*	intergenic	LSC
InDel	1	A	*trnG-trnR*	intergenic	LSC
InDel	1	A	*rpoB-trnC*	intergenic	LSC
InDel	1	A	*rps18-rpl20*	intergenic	LSC
InDel	1	A	*petB-petD*	intergenic	LSC
InDel	1	A	*petD-rpoA*	intergenic	LSC
InDel	1	A	*ndhA intron*	intron	SSC
InDel	1	T	*rps19-rpl2*	intergenic	IR
InDel	1	T	*petN-psbM*	intergenic	LSC
InDel	1	T	*ndhC-trnV*	intergenic	LSC
InDel	1	T	*atpB-rbcL*	intergenic	LSC
InDel	1	T	*rpl20-rps12*	intergenic	LSC
InDel	1	T	*rpl20-rps12*	intergenic	LSC
InDel	1	T	*ccsA-ndhD*	intergenic	SSC
InDel	1	T	*atpF intron*	intron	LCS
InDel	1	T	*atpF intron*	intron	LCS
InDel	1	T	*ycf3 intron*	intron	LSC
InDel	1	T	*clpP intron*	intron	LSC
InDel	2	AA	*rpl2-trnH*	intergenic	IR
InDel	2	TG	*rpl16 intron*	intron	LSC
InDel	4	TGAA	*ndhF-trnL*	intergenic	SSC
InDel	7	TTATATT	*trnK-rps16*	intergenic	LSC
InDel	9	CGGTGTAAT	*rpoC2*	gene	LSC
InDel	13	GAAAAAAAAAAAA	*rps2-rpoC4*	intergenic	LSC
InDel	13	TTCTTTTTTTTTT	*rps2-rpoC2*	intergenic	LSC

InDel	14	TATAATATAATTAT	*psbZ-trnG*	intergenic	LSC
InDel	14	TTATTTATTATTTA	*atpH-atpI*	intergenic	LSC
InDel	20	AAAATTAAAAAATTTCAATT	*petA-psbJ*	intergenic	LSC
InDel	83	(TTTAATTTAACTATTAACTTTATTT AATTTAACTATTAACTTTA)x2	*petN-psbM*	intergenic	LSC
Ts	1	C→T	*rpl2 intron*	intergenic	IR
Ts	1	C→T	*trnL-ndhB*	intergenic	IR
Ts	1	C→T	*psaJ-rpl33*	intergenic	LSC
Ts	1	G→A	*trnL-trnF*	intergenic	LSC
Ts	1	G→A	*ndhG-ndhI*	intergenic	SSC
Ts	1	T→C	*rpl36-infA*	intergenic	LSC
Ts; N	1	C→T	*ndhH*	gene	SSC
Ts; N	1	G→A	*rpoC1*	gene	LSC
Ts; N	1	G→A	*rpoC1*	gene	LSC
Ts; N	1	T→C	*rpoA*	gene	LSC
Ts; S	1	A→G	*ycf1*	gene	SSC
Ts; S	1	T→C	*rps14*	gene	LSC
Ts; S	1	T→C	*psaB*	gene	LSC
Ts; S	1	T→C	*accD*	gene	LSC
Ts; S	1	T→C	*ycf1*	gene	SSC
Tv	1	A→T	*trnK-rps16*	intergenic	LSC
Tv	1	A→T	*rps2-rpoC3*	intergenic	LSC
Tv	1	A→T	*petD-rpoA*	intergenic	LSC
Tv	1	A→T	*petD-rpoA*	intergenic	LSC
Tv	1	A→T	*ndhF-trnL*	intergenic	SSC
Tv	1	A→T	*ndhF-trnL*	intergenic	SSC
Tv	1	C→G	*trnP-psaJ*	intergenic	LSC
Tv	1	G→C	*petD-rpoA*	intergenic	LSC
Tv	1	G→T	*trnS-trnG*	intergenic	LSC
Tv	1	G→T	*rps2-rpoC3*	intergenic	LSC
Tv	1	G→T	*rpoB-trnC*	intergenic	LSC

Tv	1	G→T	*trnL-ccsA*	intergenic	SSC
Tv	1	G→T	*ndhI-ndhA*	intergenic	SSC
Tv	1	G→T	*trnG*	non-translate	LSC
Tv	1	T→A	*rps2-rpoC3*	intergenic	LSC
Tv	1	T→A	*ndhD-psaC*	intergenic	SSC
Tv	1	T→G	*trnE-trnG*	intergenic	LSC
Tv; N	1	A→T	*ycf1*	gene	SSC
Tv; N	1	C→A	*psaA*	gene	LSC
Tv; N	1	C→A	*ndhG*	gene	SSC
Tv; N	1	G→T	*rpl22*	gene	LSC
Tv; N	1	T→G	*ycf1*	gene	SSC

Table S8. Phylogenetic studies that support each one of the three possible topologies among the COM clade.

Inferences made based on only one gene were not included. Internal node support correspond to Boostrap values except for the ones indicated with JK (jackknife support) or PP (Posterior Probability).

Publication	No. genes	Genome	Support value for the internal node
		(C(O,M)) topology	
Davis & Wurdack, 2004	3	mitochondrial, nuclear	52
Zhu et al., 2007	4	plastid, mitochondrial, nuclear	<50
Wang et al., 2009	12	plastid, nuclear	<50
Wurdack & Davis, 2009	13	plastid, mitochondrial, nuclear	86/97
Qiu et al., 2010	4	mitochondrial	97
Soltis et al., 2011	17	plastid, mitochondrial, nuclear	59
Ruhfel et al., 2014	78	plastid (1,2 codon positions)	52
Wu et al., 2014	79	plastid	48
Sun et al., 2015	4	mitochondrial	83
Sun et al., 2016	4	plastid, mitochondrial	<50
		((C,O)M) topology	
Nickrent, Der & Anderson, 2005	4	plastid, mitochondrial, nuclear	56
Moore et al., 2010	87	plastid	88
Ruhfel et al., 2014	78	plastid	91
Sun et al., 2015	78	plastid	81
Chen et al., 2016	5	plastid, mitochondrial	44
Jo et al., 2016	80	plastid	100
Cauz-Santos et al., 2017	43	plastid	87
Gitzendanner et al., 2018	78	plastid (amino acids)	59
		((C,M)O) topology	
Zhang & Simmons, 2006	7	plastid, nuclear	87JK
Soltis, Gitzendanner & Soltis, 2007	3	plastid, nuclear	54PP
Burleigh, Hilu & Soltis, 2009	5	plastid, nuclear	58
Moore et al., 2011	(IR)	plastid	49
Li et al., 2019	80	plastid	85

Fig. S1. Plastid genome alignment from Mauve among selected COM representatives.

One IR was removed in all species. For each sequence, the first line shows the collinear blocks, the second line represents the plastid sequence with white boxes for protein-coding regions, green boxes for tRNAs, red boxes for rRNAs, and pink boxes for the exact location of the IR (when available). Clade abbreviations are indicated by "M" for Malpighiales, "C" for Celastrales and "O for Oxalidales.

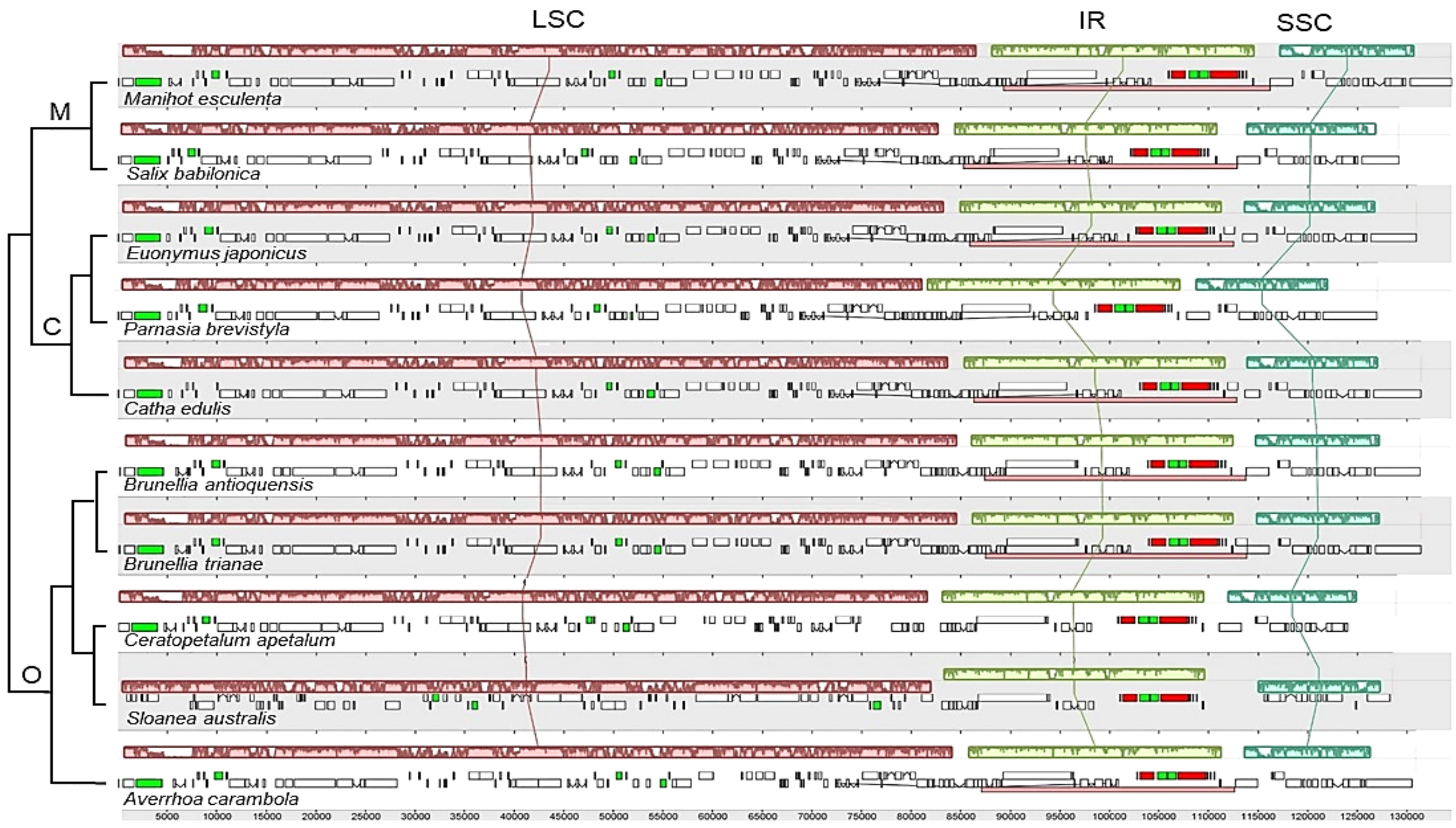

Fig. S2. Plastid genome alignment from mVISTA among all available sequences of COM species.

Reference sequence: *B. antioquiensis.* Clade abbreviations are indicated by "M" for Malpighiales, "C" for Celastrales and "O for Oxalidales.

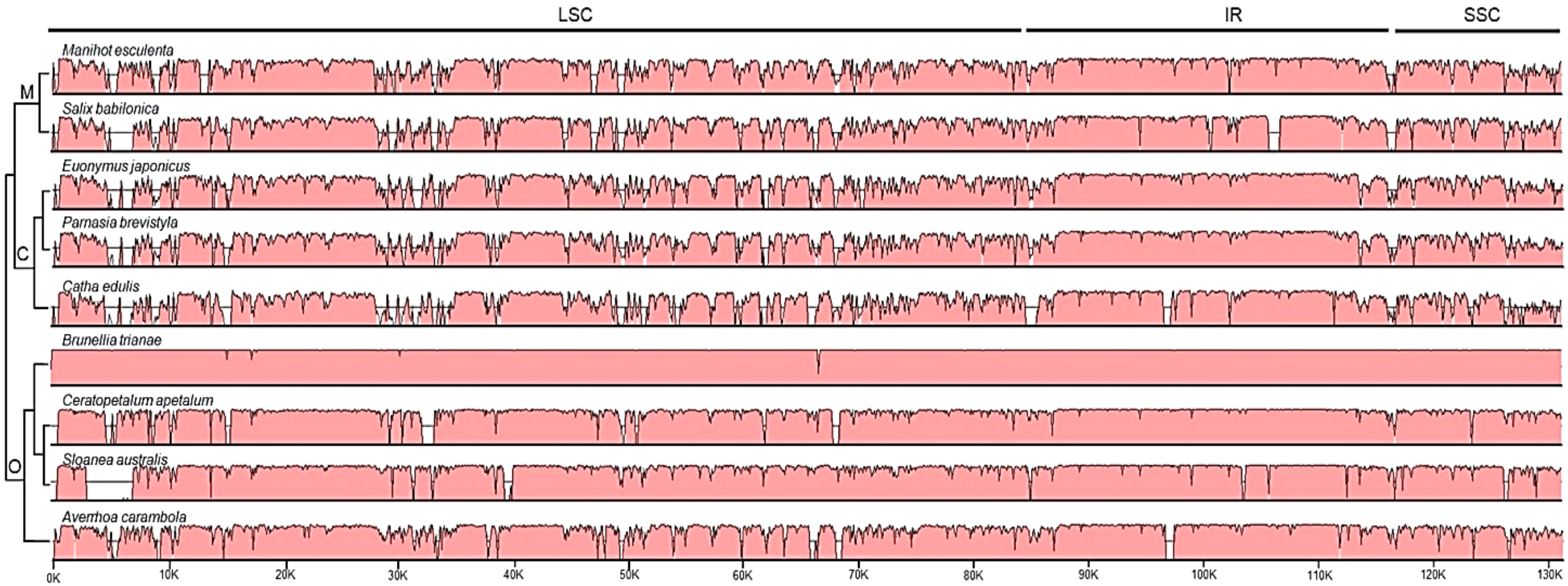

Literature cited

Asif MH, Mantri SS, Sharma A, Srivastava A, Trivedi I, Gupta P, Mohanty CS, Sawant SV, Tuli R. 2010. Complete sequence and organisation of the *Jatropha curcas* (Euphorbiaceae) chloroplast genome. *Tree Genetics & Genomes* 6:941–952. DOI: 10.1007/s11295-010-0303-0.

Bausher MG, Singh ND, Lee S-B, Jansen RK, Daniell H. 2006. The complete chloroplast genome sequence of *Citrus sinensis* (L.) Osbeck var "Ridge Pineapple": organization and phylogenetic relationships to other angiosperms. *BMC Plant Biology* 6:21. DOI: 10.1186/1471-2229-6-21.

Burleigh JG, Hilu KW, Soltis DE. 2009. Inferring phylogenies with incomplete data sets: a 5-gene, 567-taxon analysis of angiosperms. *BMC Evolutionary Biology* 9:61. DOI: 10.1186/1471-2148-9-61.

Cauz-Santos LA, Munhoz CF, Rodde N, Cauet S, Santos AA, Penha HA, Dornelas MC, Varani AM, Oliveira GCX, Bergès H, Vieira MLC. 2017. The chloroplast genome of *Passiflora edulis* (Passifloraceae) assembled from long sequence reads: Structural organization and phylogenomic studies in Malpighiales. *Frontiers in Plant Science* 8. DOI: 10.3389/fpls.2017.00334.

Chen Z-D, Yang T, Lin L, Lu L-M, Li H-L, Sun M, Liu B, Chen M, Niu Y-T, Ye J-F, Cao Z-Y, Liu H-M, Wang X-M, Wang W, Zhang J-B, Meng Z, Cao W, Li J-H, Wu S-D, Zhao H-L, Liu Z-J, Du Z-Y, Wang Q-F, Guo J, Tan X-X, Su J-X, Zhang L-J, Yang L-L, Liao Y-Y, Li M-H, Zhang G-Q, Chung S-W, Zhang J, Xiang K-L, Li R-Q, Soltis DE, Soltis PS, Zhou S-L, Ran J-H, Wang X-Q, Jin X-H, Chen Y-S, Gao T-G, Li J-H, Zhang S-Z, Lu A-M, China Phylogeny Consortium. 2016. Tree of life for the genera of Chinese vascular plants: Tree of life of Chinese vascular plants. *Journal of Systematics and Evolution* 54:277–306. DOI: 10.1111/jse.12219.

Cheng L, Huang W, Lan Y, Cao Q, Su S, Zhou Z, Wang J, Liu J, Hu G. 2018. The complete chloroplast genome sequence of the wild Chinese chestnut (*Castanea mollissima*). *Conservation Genetics Resources* 10:291–294. DOI: 10.1007/s12686-017-0805-2.

Choi KS, Park S. 2016. The complete chloroplast genome sequence of *Euonymus japonicus* (Celastraceae). *Mitochondrial DNA Part A* 27:3577–3578. DOI: 10.3109/19401736.2015.1075127.

Daniell H, Wurdack KJ, Kanagaraj A, Lee S-B, Saski C, Jansen RK. 2008. The complete nucleotide sequence of the cassava (*Manihot esculenta*) chloroplast genome and the evolution of *atpF* in Malpighiales: RNA editing and multiple losses of a group II intron. *Theoretical and Applied Genetics* 116:723. DOI: 10.1007/s00122-007-0706-y.

Davis CC, Wurdack KJ. 2004. Host-to-parasite gene transfer in flowering plants: Phylogenetic evidence from Malpighiales. *Science* 305:676–678. DOI: 10.1126/science.1100671.

Foster CSP, Sauquet H, van der Merwe M, McPherson H, Rossetto M, Ho SYW. 2016. Evaluating the impact of genomic data and priors on bayesian estimates of the Angiosperm evolutionary timescale. *Systematic Biology*:syw086. DOI: 10.1093/sysbio/syw086.

Gitzendanner MA, Soltis PS, Wong GK, Ruhfel BR, Soltis DE. 2018. Plastid phylogenomic analysis of green plants: a billion years of evolutionary history. *American Journal of Botany* 105:291–301.

Greiner S, Wang X, Herrmann RG, Rauwolf U, Mayer K, Haberer G, Meurer J. 2008. The complete nucleotide sequences of the 5 genetically distinct plastid genomes of *Oenothera*,

subsection *Oenothera*: II. A microevolutionary view using bioinformatics and formal genetic data. *Molecular Biology and Evolution* 25:2019–2030. DOI: 10.1093/molbev/msn149.

Gu C, Tembrock LR, Zheng S, Wu Z. 2018. The complete chloroplast genome of *Catha edulis*: A comparative analysis of genome features with related species. *International Journal of Molecular Sciences* 19:525. DOI: 10.3390/ijms19020525.

Jansen RK, Cai Z, Raubeson LA, Daniell H, dePamphilis CW, Leebens-Mack J, Muller KF, Guisinger-Bellian M, Haberle RC, Hansen AK, Chumley TW, Lee S-B, Peery R, McNeal JR, Kuehl JV, Boore JL. 2007. Analysis of 81 genes from 64 plastid genomes resolves relationships in angiosperms and identifies genome-scale evolutionary patterns. *Proceedings of the National Academy of Sciences* 104:19369–19374. DOI: 10.1073/pnas.0709121104.

Jansen RK, Saski C, Lee S-B, Hansen AK, Daniell H. 2011. Complete plastid genome sequences of three Rosids (*Castanea, Prunus, Theobroma*): Evidence for at least two independent transfers of *rpl22* to the nucleus. *Molecular Biology and Evolution* 28:835–847. DOI: 10.1093/molbev/msq261.

Jo S, Kim HW, Kim YK, Cheon SH, Kim K-J. 2016. Complete plastome sequence of *Averrhoa carambola* L. (Oxalidaceae). *Mitochondrial DNA Part B* 1:609–611. DOI: 10.1080/23802359.2016.1209095.

Kim H-W, Kim K-J. 2016. The complete plastome sequence of *Pentactina rupicola* Nakai (Rosaceae), a genus endemic to Korea. *Mitochondrial DNA Part B* 1:698–700. DOI: 10.1080/23802359.2016.1225523.

Li H-T, Yi T-S, Gao L-M, Ma P-F, Zhang T, Yang J-B, Gitzendanner MA, Fritsch PW, Cai J, Luo Y, Wang H, van der Bank M, Zhang S-D, Wang Q-F, Wang J, Zhang Z-R, Fu C-N, Yang J, Hollingsworth PM, Chase MW, Soltis DE, Soltis PS, Li D-Z. 2019. Origin of angiosperms and the puzzle of the Jurassic gap. *Nature Plants* 5:461–470. DOI: 10.1038/s41477-019-0421-0.

Mao Q, Bi G. 2016. Complete chloroplast genome of *Ficus racemosa* (Moraceae). *Mitochondrial DNA Part A* 27:4655–4656. DOI: 10.3109/19401736.2015.1106488.

Melodelima C, Lobréaux S. 2013. Complete *Arabis alpina* chloroplast genome sequence and insight into its polymorphism. *Meta Gene* 1:65–75. DOI: 10.1016/j.mgene.2013.10.004.

Moore MJ, Hassan N, Gitzendanner MA, Bruenn RA, Croley M, Vandeventer A, Horn JW, Dhingra A, Brockington SF, Latvis M, Ramdial J, Alexandre R, Piedrahita A, Xi Z, Davis CC, Soltis PS, Soltis DE. 2011. Phylogenetic analysis of the plastid inverted repeat for 244 species: Insights into deeper-level angiosperm relationships from a long, slowly evolving sequence region. *International Journal of Plant Sciences* 172:541–558. DOI: 10.1086/658923.

Moore MJ, Soltis PS, Bell CD, Burleigh JG, Soltis DE. 2010. Phylogenetic analysis of 83 plastid genes further resolves the early diversification of eudicots. *Proceedings of the National Academy of Sciences* 107:4623–4628. DOI: 10.1073/pnas.0907801107.

Nickrent DL, Der JP, Anderson FE. 2005. Discovery of the photosynthetic relatives of the" Maltese mushroom" *Cynomorium*. *BMC Evolutionary Biology* 5:38.

Okumura S, Sawada M, Park YW, Hayashi T, Shimamura M, Takase H, Tomizawa K-I. 2006. Transformation of poplar (*Populus alba*) plastids and expression of foreign proteins in tree chloroplasts. *Transgenic Research* 15:637–646. DOI: 10.1007/s11248-006-9009-3.

Paiva JA, Prat E, Vautrin S, Santos MD, San-Clemente H, Brommonschenkel S, Fonseca PG, Grattapaglia D, Song X, Ammiraju JS, Kudrna D, Wing RA, Freitas AT, Bergès H, Grima-Pettenati J. 2011. Advancing *Eucalyptus* genomics: identification and sequencing of lignin biosynthesis genes from deep-coverage BAC libraries. *BMC Genomics* 12. DOI: 10.1186/1471-2164-12-137.

Qiu Y-L, Li L, Wang B, Xue J-Y, Hendry TA, Li R-Q, Brown JW, Liu Y, Hudson GT, Chen Z-D. 2010. Angiosperm phylogeny inferred from sequences of four mitochondrial genes. *Journal of Systematics and Evolution* 48:391–425. DOI: 10.1111/j.1759-6831.2010.00097.x.

Ravi V, Khurana JP, Tyagi AK, Khurana P. 2006. The chloroplast genome of mulberry: complete nucleotide sequence, gene organization and comparative analysis. *Tree Genetics & Genomes* 3:49–59. DOI: 10.1007/s11295-006-0051-3.

Rivarola M, Foster JT, Chan AP, Williams AL, Rice DW, Liu X, Melake-Berhan A, Huot Creasy H, Puiu D, Rosovitz MJ, Khouri HM, Beckstrom-Sternberg SM, Allan GJ, Keim P, Ravel J, Rabinowicz PD. 2011. Castor bean organelle genome sequencing and worldwide genetic diversity analysis. *PLoS ONE* 6:e21743. DOI: 10.1371/journal.pone.0021743.

Rodríguez-Moreno L, González VM, Benjak A, Martí MC, Puigdomènech P, Aranda MA, Garcia-Mas J. 2011. Determination of the melon chloroplast and mitochondrial genome sequences reveals that the largest reported mitochondrial genome in plants contains a significant amount of DNA having a nuclear origin. *BMC Genomics* 12. DOI: 10.1186/1471-2164-12-424.

Ruhfel BR, Gitzendanner MA, Soltis PS, Soltis DE, Burleigh JG. 2014a. Data from: From algae to angiosperms–inferring the phylogeny of green plants (Viridiplantae) from 360 plastid genomes. DOI: 10.5061/dryad.k1t1f.

Ruhfel BR, Gitzendanner MA, Soltis PS, Soltis DE, Burleigh JG. 2014b. From algae to angiosperms–inferring the phylogeny of green plants (Viridiplantae) from 360 plastid genomes. *BMC Evolutionary Biology* 14:23.

Santos V, Almeida C, Santos V, Almeida C. 2019. The complete chloroplast genome sequences of three *Spondias* species reveal close relationship among the species. *Genetics and Molecular Biology* 42:132–138. DOI: 10.1590/1678-4685-gmb-2017-0265.

Saski C, Lee S-B, Daniell H, Wood TC, Tomkins J, Kim H-G, Jansen RK. 2005. Complete chloroplast genome sequence of *Glycine max* and comparative analyses with other legume genomes. *Plant Molecular Biology* 59:309–322. DOI: 10.1007/s11103-005-8882-0.

Sato S. 1999. Complete structure of the chloroplast genome of *Arabidopsis thaliana*. *DNA Research* 6:283–290. DOI: 10.1093/dnares/6.5.283.

Schwarz EN, Ruhlman TA, Sabir JSM, Hajrah NH, Alharbi NS, Al-Malki AL, Bailey CD, Jansen RK. 2015. Plastid genome sequences of legumes reveal parallel inversions and multiple losses of *rps16* in papilionoids: Parallel inversions and *rps16* losses in legumes. *Journal of Systematics and Evolution* 53:458–468. DOI: 10.1111/jse.12179.

Soltis DE, Gitzendanner MA, Soltis PS. 2007. A 567-taxon data set for angiosperms: the challenges posed by Bayesian analyses of large data sets. *International Journal of Plant Sciences* 168:137–157.

Soltis DE, Smith SA, Cellinese N, Wurdack KJ, Tank DC, Brockington SF, Refulio-Rodriguez NF, Walker JB, Moore MJ, Carlsward BS, Bell CD, Latvis M, Crawley S, Black C, Diouf D, Xi Z, Rushworth CA, Gitzendanner MA, Sytsma KJ, Qiu Y-L, Hilu KW, Davis CC, Sanderson MJ, Beaman RS, Olmstead RG, Judd WS, Donoghue MJ, Soltis PS. 2011.

Angiosperm phylogeny: 17 genes, 640 taxa. *American Journal of Botany* 98:704–730. DOI: 10.3732/ajb.1000404.

Sun Y, Moore MJ, Zhang S, Soltis PS, Soltis DE, Zhao T, Meng A, Li X, Li J, Wang H. 2016. Phylogenomic and structural analyses of 18 complete plastomes across nearly all families of early-diverging eudicots, including an angiosperm-wide analysis of IR gene content evolution. *Molecular Phylogenetics and Evolution* 96:93–101. DOI: 10.1016/j.ympev.2015.12.006.

Sun M, Soltis DE, Soltis PS, Zhu X, Burleigh JG, Chen Z. 2015. Deep phylogenetic incongruence in the angiosperm clade Rosidae. *Molecular Phylogenetics and Evolution* 83:156–166. DOI: 10.1016/j.ympev.2014.11.003.

Wang H, Moore MJ, Soltis PS, Bell CD, Brockington SF, Alexandre R, Davis CC, Latvis M, Manchester SR, Soltis DE. 2009. Rosid radiation and the rapid rise of angiosperm-dominated forests. *Proceedings of the National Academy of Sciences* 106:3853–3858.

Wang Y, Yang H. 2016. The complete chloroplast genome sequence of *Salix babylonica*. *Mitochondrial DNA Part A* 27:4683–4684. DOI: 10.3109/19401736.2015.1106501.

Weng M-L, Blazier JC, Govindu M, Jansen RK. 2014. Reconstruction of the ancestral plastid genome in Geraniaceae reveals a correlation between genome rearrangements, repeats, and nucleotide substitution rates. *Molecular Biology and Evolution* 31:645–659. DOI: 10.1093/molbev/mst257.

Weng M-L, Ruhlman TA, Jansen RK. 2017. Expansion of inverted repeat does not decrease substitution rates in *Pelargonium* plastid genomes. *New Phytologist* 214:842–851. DOI: 10.1111/nph.14375.

Wu Z, Gui S, Quan Z, Pan L, Wang S, Ke W, Liang D, Ding Y. 2014. A precise chloroplast genome of *Nelumbo nucifera* (Nelumbonaceae) evaluated with Sanger, Illumina MiSeq, and PacBio RS II sequencing platforms: insight into the plastid evolution of basal eudicots. *BMC Plant Biology* 14:289.

Wurdack KJ, Davis CC. 2009. Malpighiales phylogenetics: Gaining ground on one of the most recalcitrant clades in the angiosperm tree of life. *American Journal of Botany* 96:1551–1570. DOI: 10.3732/ajb.0800207.

Xia M, Zhang F, Rao H, Chi X, Khan G, Zhang Y, Yu J, Chen S. 2018. Complete chloroplast genome sequence of *Parnassia brevistyla* (Celastraceae) and phylogenetic analysis with related species. *Mitochondrial DNA Part B* 3:1187–1188. DOI: 10.1080/23802359.2018.1524725.

Xu Q, Xiong G, Li P, He F, Huang Y, Wang K, Li Z, Hua J. 2012. Analysis of complete nucleotide sequences of 12 *Gossypium* chloroplast genomes: Origin and evolution of allotetraploids. *PLoS ONE* 7:e37128. DOI: 10.1371/journal.pone.0037128.

Zecca G, Grassi F, Tabidze V, Pipia I, Kotorashvili A, Kotaria N, Beridze T. 2019. Dates and rates in grape's plastomes: evolution in slow motion. *Current Genetics*. DOI: 10.1007/s00294-019-01004-7.

Zhang Y-H, Huang Y, Li Z-M, Zhang S-D. 2019. Characterization of the complete chloroplast genome of the vulnerable agarwood tree, *Aquilaria yunnanensis* (Thymelaeaceae). *Conservation Genetics Resources* 11:161–164. DOI: 10.1007/s12686-018-0989-0.

Zhang L-B, Simmons MP. 2006. Phylogeny and delimitation of the Celastrales inferred from nuclear and plastid genes. *Systematic Botany* 31:122–137. DOI: 10.1600/036364406775971778.

Zhao K-K, Wang J-H, Cai Y-C, Zhu Z-X, López-Pujol J, Wang H-F. 2018. Complete chloroplast genome sequence of *Heritiera angustata* (Malvaceae): an endangered plant species. *Mitochondrial DNA Part B* 3:141–142. DOI: 10.1080/23802359.2017.1422398.

Zhu X-Y, Chase MW, Qiu Y-L, Kong H-Z, Dilcher DL, Li J-H, Chen Z-D. 2007. Mitochondrial *matR* sequences help to resolve deep phylogenetic relationships in rosids. *BMC Evolutionary Biology* 7:217. DOI: 10.1186/1471-2148-7-217.
